# Supplementary material for: Nitrogen-Doped Carbon Quantum Dots for Biosensing Applications: The Effect of the Thermal Treatments on Electrochemical and Optical Properties
Source: Molecules. 2022 Dec 22;28(1):72. doi: 10.3390/molecules28010072 (PMC9821838; doi:10.3390/molecules28010072)
Supplement: Supplementary file 1 [file molecules-28-00072-s001.zip › molecules-2084543-supplementary.pdf]

**Nitrogen-doped Carbon Quantum Dots for Biosensing applications: The Effect of The Thermal Treatments on Electrochemical and Optical Properties**

Francesco Ghezzi [1], Riccardo Donnini [2], Antonio Sansonetti [3], Umberto Giovanella [4], Barbara La Ferla [5], BarbaraVercelli [2,*]

[1] *Istituto per la Scienza e la Tecnologia dei Plasmi, CNR-ISTP, Via Cozzi 53, 20125 Milano (Italy).*

[2] *Istituto di Chimica della Materia Condensata e di Tecnologie per l'Energia, CNR-ICMATE, Via Cozzi 53, 20125 Milano (Italy).*

[3] *Istituto di Scienze del Patrimonio Culturale, CNR-ISPC, Via Cozzi 53, 20125 Milano (Italy).*

[4] *Istituto di Scienze e Tecnologie Chimiche 'Giulio Natta', CNR-SCITEC, Via Alfonso Corti 12, 20133 Milano (Italy)*

[5] *Dipartimento di Biotecnologie e Bioscienze, Università degli Studi di Milano-Bicocca, Piazza della Scienza 2, 20126 Milano (Italy)*

**<u>Contents</u>**

**Table S1** **page S02**

**Figure S1** **page S03**

**Figure S2** **page S04**

**Figure S3** **page S05**

**Table S1** – Fitting parameters, intercept ($E_i$) and slope, of the peak potentials versus pH of N-CQDs-160-8.

| **pH range** | **$E_{i(ox)}$/V** | **Slope/mV** | **$E_{i(red)}$/V** | **Slope/mV** |
|---|---|---|---|---|
| **1-3** | 0.43 | -55 | 0.02 | -61 |
| **5-12** | 0.17 | 25 | 0.2 | -126 |

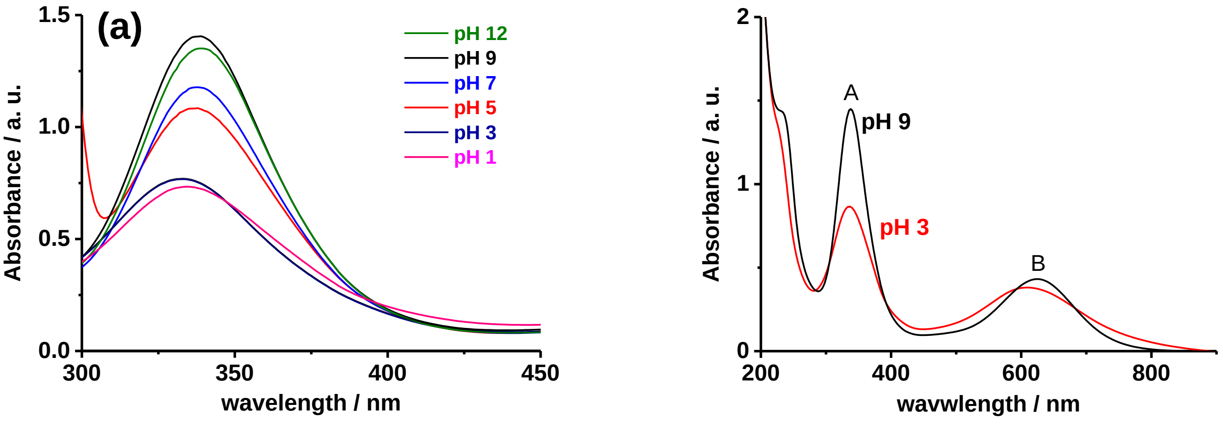


**Figure S1** – (a) UV-vis spectra of 0.5 mg ml$^{-1}$ N-CQDs-160-8 solutions in different pH buffers. (b) UV-vis spectrum of 0.5 mg ml$^{-1}$ N-CQDs-160-8 solutions at the pH range 3 (red line) and 9 (black line).

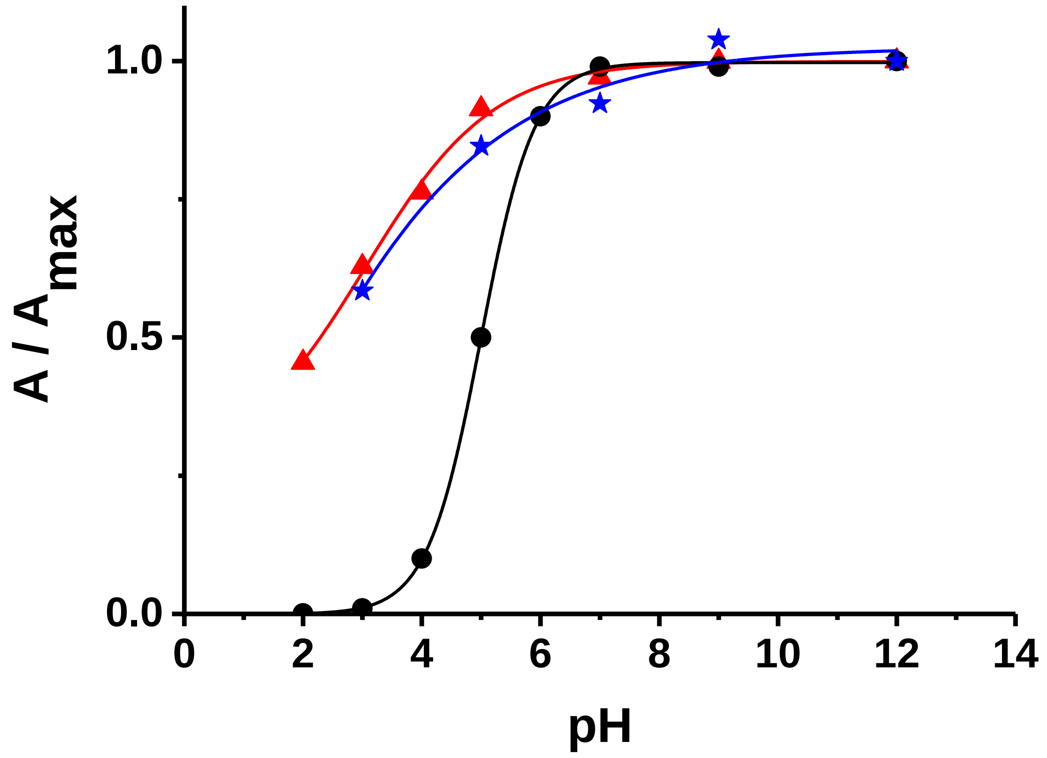


**Figure S2** – Dependence vs pH of the un-protonated pyridine N-atom ($\frac{A}{A_{max}}$) of samples N-CQDs-160-1 (red line, triangles), N-CQDs-160-8 (blue line, stars) and theoretical curve (black line, dots).

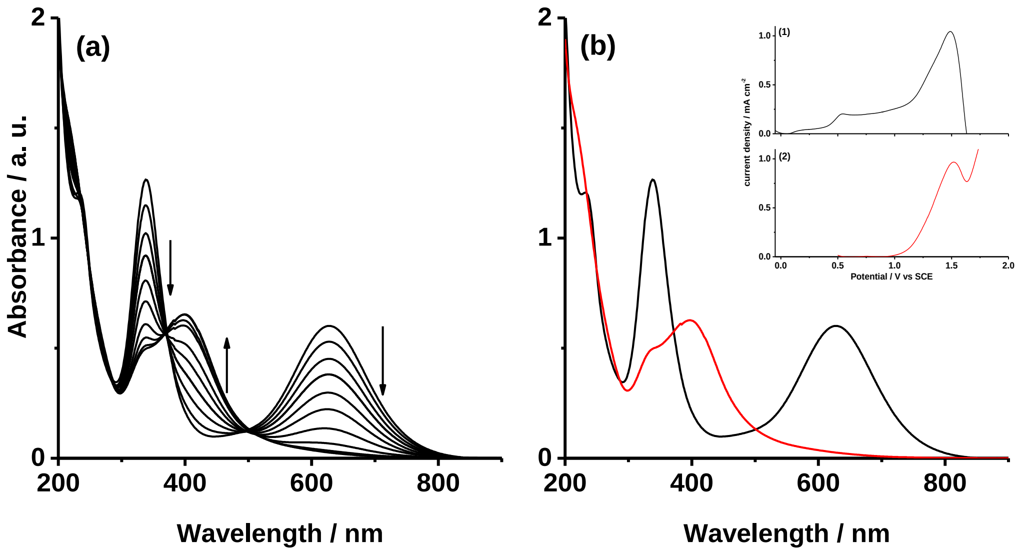


**Figure S3** – a) UV-vis spectra of 0.5 mg ml$^{-1}$ N-CQDs-160-8 solution at increasing charge. Applied potential 0.8 V vs SCE. Electrolytic medium 0.1 M NaClO4/pH 9 buffer. b) UV-vis spectrum of N-CQDs-160-8 (black line) and of NO-CQDs-160-8 (red line). Inset: single sweep cyclic voltammograms of N-CQDs-160-8 (1) and NO-CQDs-160-8 (2).
